# Rheological Characterization and Printability of Sodium Alginate–Gelatin Hydrogel for 3D Cultures and Bioprinting

**DOI:** 10.3390/biomimetics10010028

**Published:** 2025-01-04

**Authors:** Mohan Kumar Dey, Ram V. Devireddy

**Affiliations:** Bioengineering Laboratory, Department of Mechanical Engineering, Louisiana State University, Baton Rouge, LA 70803, USA; mdey1@lsu.edu

**Keywords:** bioprinting, hydrogel, alginate, gelatin, rheological characterization

## Abstract

The development of biocompatible hydrogels for 3D bioprinting is essential for creating functional tissue models and advancing preclinical drug testing. This study investigates the formulation, printability, mechanical properties, and biocompatibility of a novel Alg-Gel hydrogel blend (alginate and gelatin) for use in extrusion-based 3D bioprinting. A range of hydrogel compositions were evaluated for their rheological behavior, including shear-thinning properties, storage modulus, and compressive modulus, which are crucial for maintaining structural integrity during printing and supporting cell viability. The printability assessment of the 7% alginate–8% gelatin hydrogel demonstrated that the 27T tapered needle achieved the highest normalized Printability Index (POI_normalized_ = 1), offering the narrowest strand width (0.56 ± 0.02 mm) and the highest printing accuracy (97.2%) at the lowest printing pressure (30 psi). In contrast, the 30R needle, with the smallest inner diameter (0.152 mm) and highest printing pressure (80 psi), resulted in the widest strand width (0.70 ± 0.01 mm) and the lowest accuracy (88.8%), resulting in a POI_normalized_ of 0.274. The 30T and 27R needles demonstrated moderate performance, with POI_normalized_ values of 0.758 and 0.558, respectively. The optimized 7% alginate and 8% gelatin blend demonstrated favorable printability, mechanical strength, and cell compatibility with MDA-MB-213 breast cancer cells, exhibiting high cell proliferation rates and minimal cytotoxicity over a 2-week culture period. This formulation offers a balanced approach, providing sufficient viscosity for precision printing while minimizing shear stress to preserve cell health. This work lays the groundwork for future advancements in bioprinted cancer models, contributing to the development of more effective tools for drug screening and personalized medicine.

## 1. Introduction

Breast cancer remains the most prevalent malignancy and the leading cause of cancer-related mortality among women globally, with its high mortality rate largely attributed to its propensity for metastasis [1]. Notably, over 70% of patients with breast cancer experience bone metastasis, leading to severe complications such as bone pain, pathological fractures, nerve compression, and hypercalcemia, all of which contribute to a poor clinical prognosis [2]. The metastatic cascade of breast cancer involves a complex sequence of events, including intravasation into the bloodstream or lymphatic system, translocation to distal bone tissues, extravasation into the bone microenvironment, and eventual colonization to form secondary tumors [3]. Understanding this metastatic process is critical for the development of novel therapeutic strategies aimed at improving patient survival.

Traditionally, 2D cell culture and animal models have been the mainstays for studying cancer metastasis and drug discovery [4]. However, these approaches lack the ability to fully replicate the dynamic and complex tumor microenvironment. While advancements in 3D bioprinting address some of these challenges, current hydrogels often suffer from limitations such as insufficient mechanical stability, poor nutrient diffusion, and a lack of bioactive cues necessary for faithfully mimicking the bone microenvironment involved in metastasis [5]. However, these models present significant limitations. Two-dimensional cell cultures fail to replicate the three-dimensional tumor microenvironment essential for accurate modeling of metastatic processes [6]. Although animal models provide a more natural environment, they are constrained by ethical concerns, high costs, time-intensive procedures, and issues with reproducibility. These shortcomings hinder the reliability of drug testing and highlight the need for more advanced and physiologically relevant in vitro models that can bridge the gap between in vitro and in vivo studies [7,8].

To address these challenges, three-dimensional (3D) tumor models have been developed, offering a more accurate representation of the physiological cell–cell and cell–matrix interactions that occur in vivo [9]. Among the various bio-fabrication techniques for constructing 3D tumor tissues, 3D bioprinting has emerged as a promising approach due to its ability to precisely and rapidly fabricate complex tissue mimics [10]. Despite its promise, challenges remain, including achieving uniform cell distribution, maintaining hydrogel fidelity over time, and replicating the heterogeneity of native tumor environments. Unlike conventional methods that involve printing cell-free scaffolds followed by cell seeding, 3D bioprinting enables the accurate spatial arrangement of multiple cell types within biomaterials (bioinks), thereby creating a more biomimetic microenvironment for in vitro tumor tissue models [11].

A critical component in 3D bioprinting is the choice of bioink, which must not only exhibit good printability but also provide a microenvironment that closely resembles in vivo conditions. Gelatin methacrylate (GelMA), a collagen derivative, has gained popularity as a bioink due to its tunable mechanical properties, rapid crosslinking capability, and retention of collagen’s bioactive features [12]. However, GelMA-based hydrogels often require optimization to address limitations such as their limited long-term mechanical stability and potential for incomplete crosslinking, which may affect their ability to support cell behavior in complex metastatic models [5,13]. GelMA, often used in combination with other materials such as hyaluronan methacrylate (HAMA) [14] and hydroxyapatite nanoparticles (HAPs) [15], enables the creation of biomimetic tissue structures that support cell attachment, promote angiogenesis, and mimic the extracellular matrix (ECM) of bone tissue [16].

In this study, we present a three-dimensional bioprinted scaffold composed of sodium alginate and gelatin for cancer cell culture. The scaffold demonstrates biocompatibility with cancer cells, enabling sustained cell growth and viability within the 3D matrix. This platform provides a physiologically relevant environment that mimics in vivo conditions, thereby facilitating the study of cancer cell behavior, including metastasis. Additionally, the scaffold serves as a robust model for evaluating the efficacy of anti-cancer therapeutics, offering valuable insights for drug development and personalized medicine applications. This approach promises to advance cancer research by providing an accessible and reproducible tool for in vitro studies.

## 2. Materials and Methods

### 2.1. Preparation of Hydrogel

Alg-Gel hydrogels were prepared following a modified protocol based on previous studies [17,18]. For a 7% Alg–8% Gel blend, gelatin was first dissolved in 1× PBS at a concentration of 0.06 g/mL at 60 °C. Sodium alginate was then added at a concentration of 0.05 g/mL, and the solution was stirred continuously for 1 h to ensure homogeneity [19]. Three additional Alg-Gel blend ratios (4:8, 5:6, and 6:4) were prepared to evaluate the structural deformation and printing fidelity across different compositions, as shown in Table 1. The prepared mixtures were then transferred to sterile syringes and stored at −25 °C overnight, promoting gelation to enhance viscosity, structural integrity, and print fidelity during bioprinting. Prior to printing, the hydrogels were thawed in a 37 °C thermal bath and centrifuged at 1000 rpm for 1 min to remove any air bubbles.

Alginic acid sodium salt (low viscosity; 1% solution in water at 20 °C), calcium chloride (CaCl_2_; white powder; 10% solution in water), and phosphate-buffered saline (PBS; pH 7.4, 1X) were obtained from ThermoFisher SCIENTIFIC, Waltham, MA, USA. Type A gelatin (derived from porcine skin) was procured from MP Biomedicals, Santa Ana, CA, USA.

### 2.2. Scaffold Design

**Figure 1:**
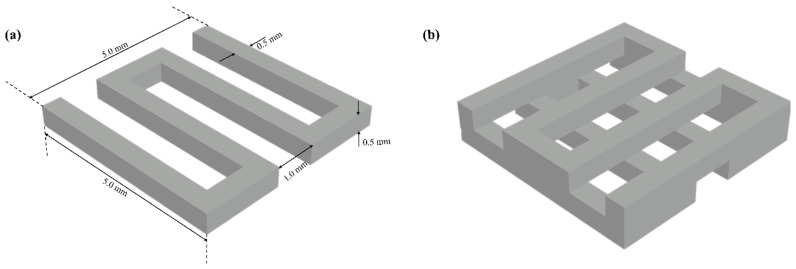
(**a**) Scaffold grid design with extruded square along the path line and (**b**) layered scaffold configuration with 90° rotation and z-axis duplication.

The scaffold model was designed in AutoCAD with a grid pattern measuring 5 mm × 5 mm × 1 mm (L × W × H). To create the grid, a path line was drawn, and a 0.5 mm square was positioned so that its center aligned with the center of the path line, which was then extruded as shown in Figure 1a. The extruded pattern was subsequently rotated to 90 degrees and duplicated along the z-axis with a 0.5 mm offset to achieve the desired layered grid configuration. The resulting scaffold grid pattern is shown in Figure 1b.

### 2.3. Rheological Characterization Testing

**Figure 2:**
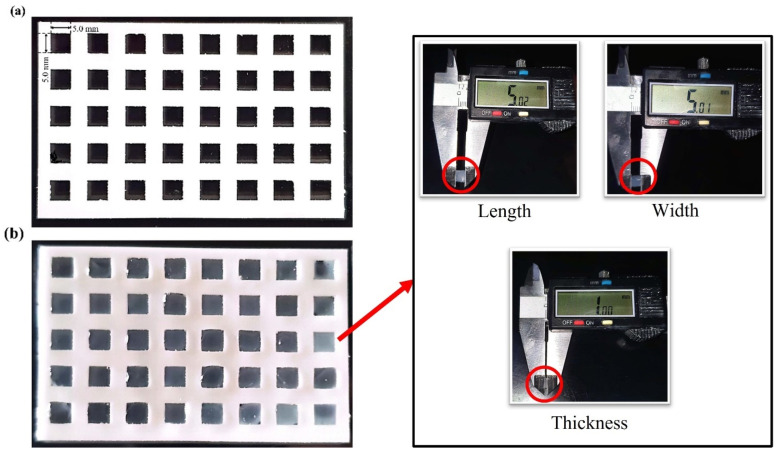
(**a**) Laser-cut square-shaped molds and (**b**) casting process for Alg-Gel hydrogel samples.

Square-shaped molds (5 mm × 5 mm × 1 mm) were laser-cut from a 1 mm thick plastic sheet sourced from McMaster-Carr, Los Angeles, CA, USA. Alg-Gel hydrogel blend samples were prepared using a casting approach to ensure uniformity and consistency across the samples. Laser-cut molding was preferred over 3D printing to eliminate potential variations associated with the printing process, thereby facilitating more accurate measurement of the material’s rheological properties. The mold and casting are shown in Figure 2. Following the molding process, the samples were crosslinked in a 100 mM CaCl_2_ solution, rinsed, and equilibrated in phosphate-buffered saline (PBS) for 30 min to remove any excess calcium ions. The final dimensions of the samples were measured using digital calipers.

Rheological testing was performed using a Discovery HR-2 rheometer (TA Instruments, Eden Prairie, MN, USA) equipped with an 8 mm flat plate setup to measure the storage modulus (G′) and loss modulus (G″) over an oscillatory frequency sweep ranging from 1 to 100 Hz at a strain of 1%. Additionally, compression tests were conducted from 0% to 20% under oscillatory time conditions at a strain amplitude of 1% and a frequency of 1 Hz. To assess the shear-thinning behavior, an essential characteristic for the bioprinting performance of bioinks, shear viscosity tests were carried out using the same rheometer. The shear rate was increased from 1 to 100 s^−1^ at 25 °C, utilizing a 25 mm flat plate. For each test, ten samples were used and their average and standard deviation values calculated. These tests provided valuable insights into the viscosity profile and flow characteristics of the bioink, which are crucial for optimizing print quality and fidelity.

### 2.4. Bioprinting Fidelity

Bioprinting was conducted using a commercial extrusion-based 3D bioprinter (Allevi 3.0) equipped with a syringe containing bioink. Allevi’s online bioprinting software facilitated the setup of user-defined parameters such as temperature, speed, pressure, and nozzle diameter to generate an optimized print path, which was then converted into G-Code. This G-Code precisely controlled the printer’s movements, allowing for consistent extrusion and accurate, layer-by-layer construction of complex geometries, ensuring fidelity in printed structures for cell and tissue applications. In this experiment, all printing took place at room temperature (25 °C) on the printing bed, with the extruder maintained at 25 °C and 35 °C. Printing was performed with 30- and 27-gauge regular (30R, 27R) and tapered (30T, 27T) nozzles, a set speed of 6 mm/s, and a pneumatic pressure range of 30 to 80 psi to maintain uniformity and continuity of extruded microfilaments.

Post-printing, each construction was imaged using a 12-megapixel camera, and ImageJ software (NIH, Bethesda, MD, USA) was utilized to measure printed dimensions. The printed area, *A_p_* (X mm^2^), was compared to the designed area, *A_d_* (16 mm^2^), to calculate the percentage printing accuracy for each sample. The printing accuracy was determined using the following equation [19]. Each printability assessment used three samples per bioink blend to ensure reliability.

(1) $$Printing\;Accuracy\left(\%\right)=\left[1-\frac{\left|A_{p}-A_{d}\right|}{A_{d}}\right]\times 100$$

### 2.5. Cell Viability and Compatibility with 3D-Bioprinted Scaffolds

**Figure 3:**
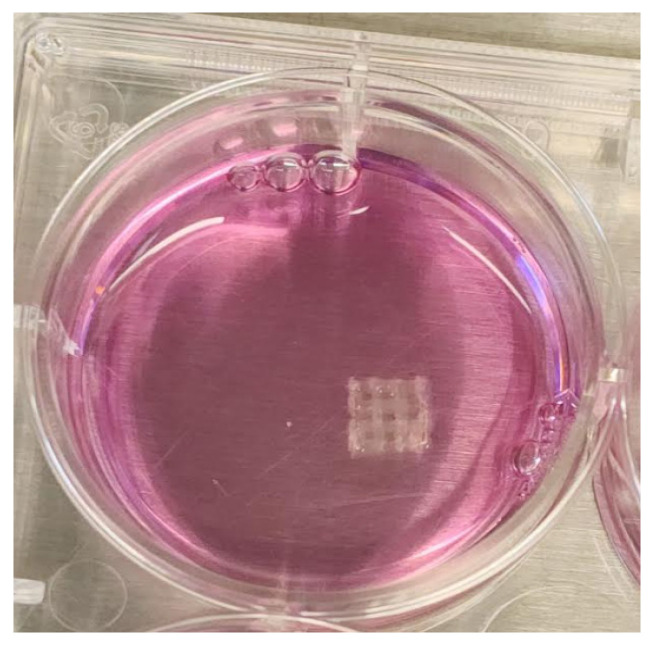
MDA-MB-231 cells seeded on 3D-bioprinted scaffolds in a 12-well plate.

MDA-MB-213 cells were purchased from ATCC (American Type Culture Collection, Biotechnology Research, Manassas, VA, USA). Cells were cultured in DMEM medium supplemented with 10% (*v*/*v*) fetal bovine serum (FBS) and 1% (*v*/*v*) penicillin/streptomycin. They were maintained at 37 °C in a humidified incubator with 5% CO_2_ and 95% relative humidity. After the initial culture, cells were detached and seeded at a density of 30,000 cells per well in a 12-well plate, where they were grown on the 3D-bioprinted scaffolds as shown in Figure 3. The same culture medium was used to support cell growth on the scaffold.

To assess cell viability on the bioprinted scaffolds, we employed the Invitrogen™ LIVE/DEAD™ Cell Imaging Kit (488/570), ThermoFisher SCIENTIFIC, Waltham, MA, USA, a sensitive two-color fluorescence assay optimized for FITC and Texas Red™ filters. Live and dead cell imaging was performed at intervals of 1 day, 1 week, and 2 weeks using a Nikon Eclipse Ti2 fluorescence microscope, Melville, NY, USA.

## 3. Results and Discussion

### 3.1. Rheological Characterization of Alg-Gel Hydrogels

**Figure 4:**
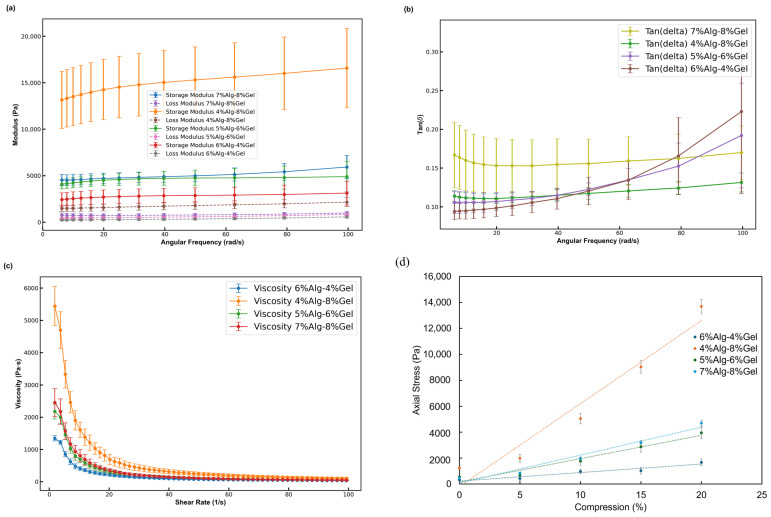
Rheological characterization of hydrogel mixtures with varying alginate and gelatin concentrations (4% Alg–8% Gel, 5% Alg–6% Gel, 5% Alg–6% Gel, 7% Alg–8% Gel). (**a**) Storage modulus (G′) and loss modulus (G″) as a function of angular frequency, showing an increase in both moduli with higher alginate concentration; (**b**) tan δ vs. angular frequency for the hydrogel mixtures, with tan δ values consistently below 1 across all formulations; (**c**) shear viscosity as a function of shear rate, demonstrating shear-thinning behavior in all hydrogel mixtures. (**d**) Axial stress vs. compression percentage, highlighting distinct mechanical behaviors across formulations, with 4% Alg–8% Gel showing the highest compressive strength.

The rheological properties of four hydrogel formulations with distinct alginate (Alg) and gelatin (Gel) concentrations (6% Alg–4% Gel, 4% Alg–8% Gel, 5% Alg–6% Gel, and 7% Alg–8% Gel) were analyzed to assess their viscoelastic behavior after crosslinking with CaCl_2_. At room temperature (25 °C), all formulations demonstrated a higher storage modulus (G′) than loss modulus (G″), indicating predominantly elastic behavior. As illustrated in Figure 4a, at an angular frequency of 20 rad/s, the storage modulus for the 6% Alg–4% Gel, 4% Alg–8% Gel, 5% Alg–6% Gel, and 7% Alg–8% Gel formulations was approximately 14,300 Pa, 4500 Pa, 2700 Pa, and 4700 Pa, respectively. The corresponding loss modulus was 1580 Pa, 480 Pa, 270 Pa, and 750 Pa. Both storage and loss modulus increased with frequency, reaching values at 100 rad/s of approximately 16,600 Pa, 4900 Pa, 3150 Pa, and 5900 Pa for storage modulus and 2150 Pa, 800 Pa, 580 Pa, and 980 Pa for loss modulus in the 6% Alg–4% Gel, 4% Alg–8% Gel, 5% Alg–6% Gel, and 7% Alg–8% Gel hydrogels, respectively. Across all samples, the tan δ values remained less than 1, as shown in Figure 4b, indicating a predominantly elastic response.

The flow properties of the non-crosslinked hydrogels were also examined, given their importance for cell viability during the printing process. At 25 °C, all samples exhibited shear-thinning behavior, with viscosity decreasing as the shear rate increased. Figure 4c demonstrates that at a low shear rate of 1 s^−1^, the viscosities of the 6% Alg–4% Gel, 4% Alg–8% Gel, 5% Alg–6% Gel, and 7% Alg–8% Gel hydrogels were 5450 Pa·s, 2180 Pa·s, 1350 Pa·s, and 2460 Pa·s, respectively. At a higher shear rate of 100 s^−1^, viscosities decreased to 105 Pa·s, 485 Pa·s, 350 Pa·s, and 485 Pa·s for the 6–4%, 4–8%, 5–6%, and 7–8% hydrogels, respectively, confirming the shear-thinning behavior of these hydrogels. Furthermore, compression tests on four Alg-Gel hydrogel formulations 6% Alg–4% Gel, 4% Alg–8% Gel, 5% Alg–6% Gel, and 7% Alg–8% Gel revealed distinct mechanical behaviors across different compositions. The axial stress and standard deviation were measured at compression levels of 0%, 5%, 10%, 15%, and 20%, as shown in Figure 4d. The 4% Alg–8% Gel hydrogel exhibited the highest axial stress, reaching 13,699.20 Pa at 20% compression, indicating that higher Gel content significantly enhances compressive strength. Its compressive modulus was measured at 63.98 kPa, further confirming its superior mechanical performance. In contrast, the 6% Alg–4% Gel hydrogel displayed the lowest stress increase, with 327.35 Pa at 0% compression rising to 1669.96 Pa at 20%, and a compressive modulus of 6.58 kPa, demonstrating moderate resistance to compressive loads. The 5% Alg–6% Gel formulation reached an intermediate stress of 3955.69 Pa at 20% compression, suggesting a balanced mechanical response between the 6% Alg–4% Gel and 4% Alg–8% Gel hydrogels, with a compressive modulus of 18.00 kPa. The 7% Alg–8% Gel composition, with high Alg content alongside 8% Gel, also demonstrated enhanced compressive properties, achieving 4688.56 Pa at 20% compression and a compressive modulus of 21.56 kPa. These findings indicate that increasing Gel concentration generally improves compressive strength, while varying Alg content provides further tunability in mechanical properties. This tunability is advantageous for tailoring Alg-Gel hydrogels for applications in tissue engineering, where specific stiffness and compressibility characteristics are essential for supporting cell growth and tissue formation.

### 3.2. Swelling of Alg-Gel Hydrogels

Swelling experiments were conducted using hydrogels with a volume of 100 μL (n = 3). The samples were incubated in 1 mL of PBS at 37 °C for 0.5, 1, 2, 3, 4, and 24 h. After each incubation period, the PBS was completely removed, and the hydrogels were weighed to determine their swollen mass (m_swollen_). The samples were then lyophilized to obtain their dry mass (m_dry_). The swelling ratio was calculated using Equation (3) [20]:

(2) $$Swelling\;Ratio=\frac{m_{swollen}-m_{dry}}{m_{dry}}$$

**Figure 5:**
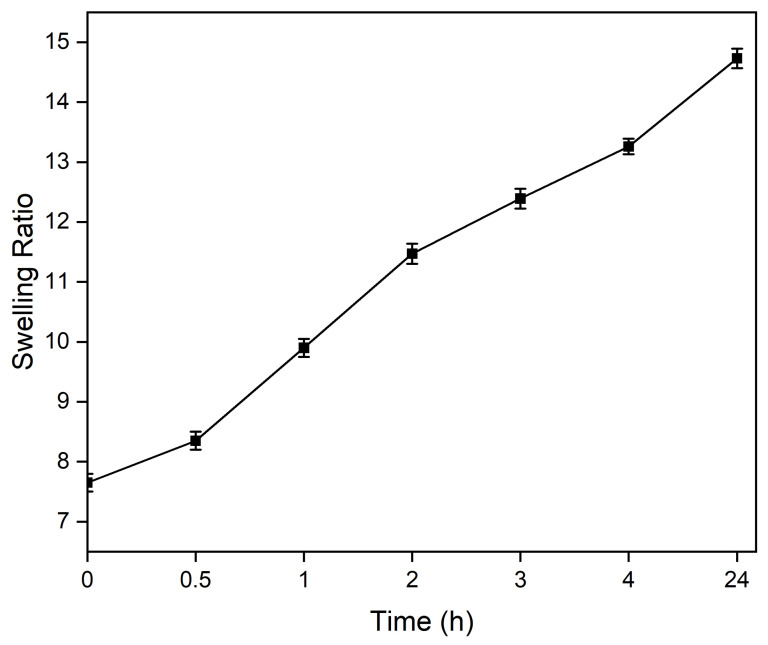
Swelling ratio of Alg-Gel hydrogels over time.

The swelling ratio of the hydrogels was assessed over various time intervals, as shown in Figure 5. The swelling ratio increased steadily with time, starting at 7.65 at 0 h and reaching a maximum of 14.73 at 24 h. Specifically, the swelling ratio increased to 8.35 at 0.5 h, 9.90 at 1 h, and 11.47 at 2 h. By 3 h, it reached 12.39, further increasing to 13.26 at 4 h, indicating a gradual equilibration process over time. These results demonstrate the hydrogels’ capacity for significant water absorption and their ability to maintain stability in a hydrated state.

### 3.3. Chemical Analysis

**Figure 6:**
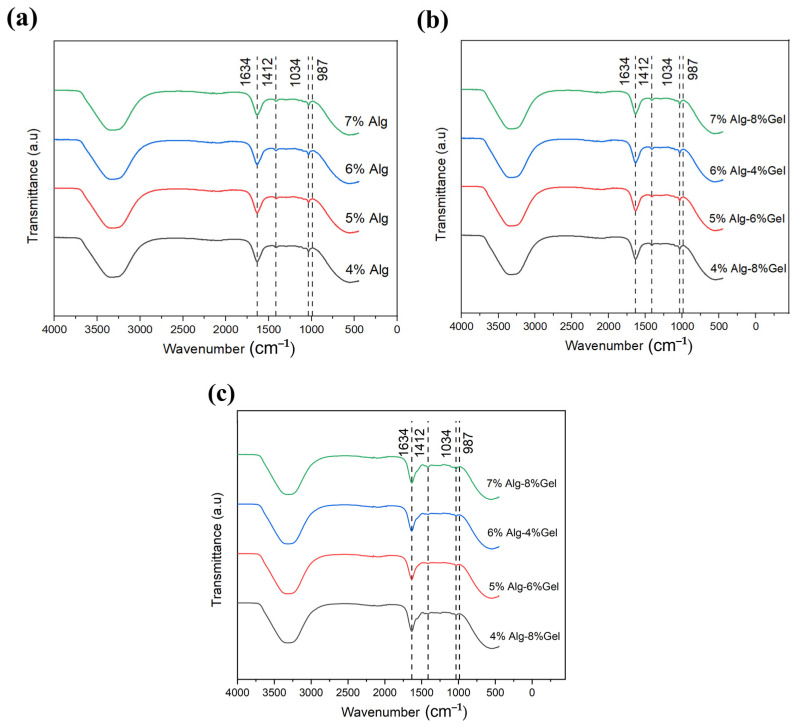
UATR spectra of (**a**) alginate, (**b**) alginate–gelatin, and (**c**) alginate–gelatin–calcium chloride.

The UATR spectra of the hydrogels, as shown in Figure 6, reveal key characteristic bands associated with alginate. These spectra were obtained using the Perkin-Elmer Spectrum Two ATR, which provides high-resolution infrared analysis, enabling the identification of functional groups and bonding characteristics within the hydrogel matrix. The observed bands confirm the presence of alginate-specific peaks, which contribute to the understanding of the molecular interactions and stability within each hydrogel composition in Figure 6a, Figure 6b, and Figure 6c, respectively. This spectroscopic analysis is essential for characterizing the chemical structure and validating the integrity of the alginate within the various Alg-Gel formulations. Notably, the peak intensities remain consistent regardless of alginate concentration. Distinct bands appear at 1034 cm^−1^, accompanied by a shoulder at 987 cm^−1^ (G-block δ(C–C)), which correspond to C–C and C–O stretching and are indicative of crosslinking within the structure. Ionic binding vibrations of the –COO– carboxylic groups in alginate are observed at 1634 cm^−1^ (asymmetric) and 1412 cm^−1^ (symmetric). These peaks shift when alginate is combined with other hydrogel components that do not participate in Ca^2+^ crosslinking. The shifting of these peaks can indicate changes in the network structure, potentially affecting the mechanical properties of the hydrogel, such as its viscosity and elasticity, which are critical for printability. The interaction of alginate with crosslinking agents like calcium ions can result in the formation of a gel network with sufficient mechanical strength for 3D printing applications.

A broad absorption band between 3300 and 3700 cm^−1^ corresponds to O–H stretching, and the C–H stretching band at approximately 2850–3000 cm^−1^ is characteristic of alginate [21,22,23,24]. In the UATR spectra of Alg hydrogels, similar alginate bands are visible along with distinct peaks characteristic of gelatin. Specifically, the amide I band appears at 1620–1630 cm^−1^, attributed to the C–O and C–N stretching in the gelatin –NH group. The amide II band is represented by a peak at 1545–1555 cm^−1^, while the amide III band, appearing at 1240–1250 cm^−1^, corresponds to C–N stretching and N–H bending, confirming the presence of gelatin [21,22,25,26]. The inclusion of gelatin contributes to the hydrogel’s overall mechanical properties, influencing both its stability and printability. The gelatin network can provide additional mechanical strength and flexibility, which is crucial for maintaining the structural integrity of 3D-printed scaffolds. With increasing gelatin concentration, these gelatin peaks intensify and shift slightly, particularly when combined with lower alginate concentrations. This shift indicates changes in the molecular interactions between the alginate and gelatin components, affecting the overall crosslinking density and the network’s ability to retain water and mechanical stability. As such, the balance between alginate and gelatin concentrations plays a critical role in determining the hydrogel’s printability and its post-printing stability, with the potential to influence cell attachment, proliferation, and differentiation on printed scaffolds. This chemical analysis highlights the significance of the interactions between alginate and gelatin and their impact on hydrogel stability and printability. The ability to fine-tune these interactions by adjusting the concentration of each component can help optimize the hydrogel’s performance for specific applications in bioprinting.

### 3.4. Printability of 7% Alg–8% Gel Hydrogel

**Figure 7:**
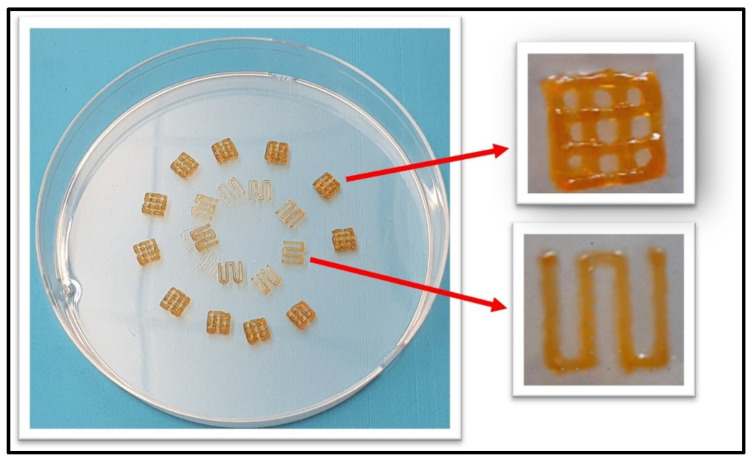
Three-dimensional bioprinting scaffold on Petri dish.

Using mechanical and chemical analysis data, we selected an optimized blend of 7% alginate and 8% gelatin for 3D bioprinting. This formulation was carefully evaluated, accounting for the increased printing pressures and the resulting shear stress on encapsulated cells. Printability testing with a range of needle sizes (see Table 2) confirmed the suitability of this blend, demonstrating that high accuracy and resolution could be reliably achieved, as shown in Figure 7, thereby validating the selection of this formulation.

To further substantiate the choice of the 7% alginate–8% gelatin blend, we employed a modified Parameter Optimization Index (POI) [27]. The POI is designed to balance high printing accuracy and compressive modulus, while minimizing printing pressure and strand width key factors in preserving cell viability and maintaining structural fidelity. Excessive printing pressures can lead to cell damage, while broader strand widths negatively impact resolution. The normalized POI, calculated using Equation (4), confirms the optimized parameters of the 7% alginate–8% gelatin blend, as presented in Table 1.

(3) $${POI}_{individual}=\frac{Printing\;Accuracy\times Compressive\;Modulus}{Printing\;Pressur\times Strand\;Width}$$

(4) $${POI}_{normalized}=\frac{{POI}_{individual}}{{POI}_{individual,max}}$$

Tapered needle 27T achieved the highest normalized Printability Index (POI_normalized_ = 1), indicating optimal performance. The 30R needle, with the smallest inner diameter (0.152 mm) and highest printing pressure (80 psi), resulted in a wider strand width (0.70 ± 0.01 mm) and the lowest printing accuracy (88.8%), leading to a POI_normalized_ of 0.274. This suggests that excessive pressure negatively impacts print fidelity. In contrast, the 30T tapered needle, with a slightly larger inner diameter (0.15 mm) and moderate pressure (35 psi), achieved a better strand width (0.63 ± 0.01 mm) and higher printing accuracy (96%), resulting in an improved POI_normalized_ of 0.758. The 27R regular needle, with a larger inner diameter (0.2 mm) and higher printing pressure (50 psi), yielded a strand width of 0.57 ± 0.02 mm, a printing accuracy of 92%, and a POI_normalized_ of 0.558, reflecting moderate performance. Finally, the 27T tapered needle, with the largest inner diameter (0.203 mm) and lowest printing pressure (30 psi), produced the narrowest strand width (0.56 ± 0.02 mm) and the highest printing accuracy (97.2%), confirming its superior printability. These results emphasize that lower printing pressures, coupled with tapered needle geometries, are crucial for optimizing hydrogel printability.

### 3.5. In Vitro Biocompatibility Assessment

**Figure 8:**
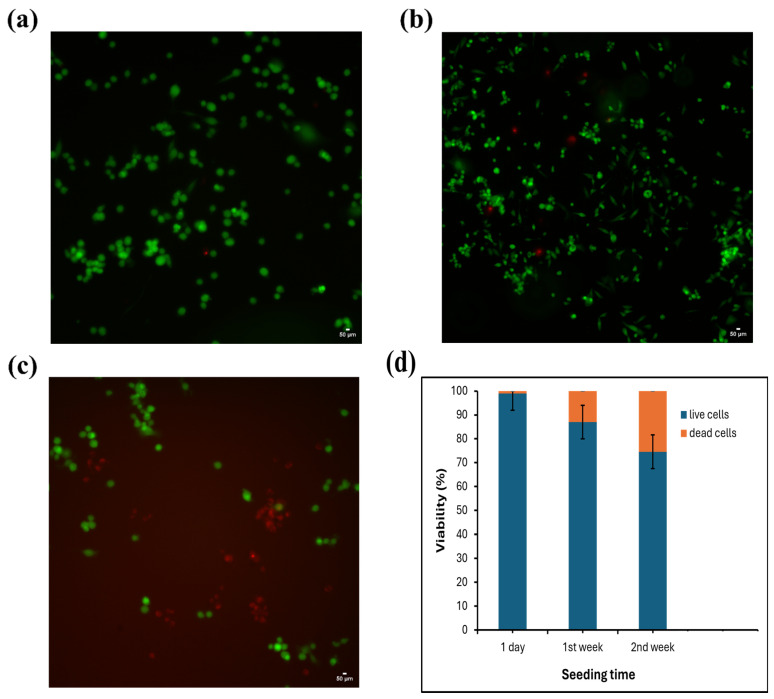
Evaluation of cell proliferation and viability of MDA-MB-213 cells cultured on Alg-Gel hydrogels over two weeks: (**a**) cell viability at 1 day; (**b**) cell viability at 1 week; (**c**) cell viability at 2 weeks, confirming hydrogel cytocompatibility and support for long-term culture; (**d**) cell viability.

The biocompatibility of the hydrogels was evaluated using MDA-MB-213 cells, with the results shown in Figure 8. Over a two-week period, the proliferative capacity of MDA-MB-213 cells generally increased across all hydrogel formulations. At the 96 h timepoint, decreasing the concentration of alginate to 2% in the Alg hydrogel group resulted in a higher cell proliferation rate of 145 ± 21%, compared to 106 ± 27% observed in the 5% Alg group [28]. This observation aligns with the known characteristics of unmodified polysaccharides like alginate, which, due to their hydrophilic nature and lack of specific binding sites, tend to inhibit cell adhesion culture within alginate-only hydrogels, which demonstrated proliferation rates lower than the cell-only control. However, modifying alginate with cell-adhesion peptides may support enhanced cell attachment, allowing for proliferation rates comparable to the cell-only control. For the 2.5% Alg–5% Gel formulation, the cell proliferation rate reached 144 ± 21%—close to the cell control rate of 151 ± 22% at 96 h. This improvement is attributed to the gelatin component, which contains sequences like GFOGER and RGD peptide motifs. These adhesive ligands enhance integrin-mediated interactions, thereby promoting cell attachment and proliferation [22,29,30].

The proliferation rates presented here are indicative rather than absolute. It is important to note that cell attachments on hydrogel surfaces can be suboptimal, with some cells detaching during washing steps. Thus, the proliferation values reflect the cells in each well that are exposed to the hydrogel, either by direct surface adhesion or growth at the well’s bottom near the hydrogel. Cell viability was assessed after 1 day, 1 week, and 2 weeks to evaluate the biocompatibility and support for cell proliferation over time, as shown in Figure 8a, Figure 8b, and Figure 8c, respectively. Notably, none of the hydrogel formulations exhibited cytotoxicity towards MDA-MB-213 cells at any of these timepoints, confirming their cytocompatibility and suitability for supporting long-term cell culture. The cell viability of MDA-MB-231 cells cultured on 3D-bioprinted scaffolds was evaluated over time. After 1 day, cell viability remained high, with 99% live cells and only 1% dead cells. By the 1st week, viability decreased to 87.02% live cells and 12.98% dead cells. By the 2nd week, a further decline in viability was observed, with 74.58% live cells and 25.42% dead cells. The results in Figure 8d indicate a gradual reduction in cell viability over time, likely due to the limitations of long-term culture conditions or scaffold properties.

## 4. Discussion

The mechanical properties of hydrogels play a crucial role in determining the accuracy of 3D-bioprinted structures and in maintaining cell viability within extrusion bioprinting. Shear-thinning behavior, an essential characteristic for printable hydrogels, allows high-viscosity formulations to flow through the nozzle without compromising structural fidelity post-extrusion. This property ensures the hydrogel maintains sufficient structural integrity upon deposition, which is advantageous for achieving precise print fidelity [31,32,33]. However, highly viscous hydrogels necessitate high extrusion pressures, increasing the risk of nozzle clogging and potentially leading to increased shear stress on embedded cells, thus compromising cell viability. Conversely, low-viscosity hydrogels, while requiring lower extrusion pressures, may spread undesirably, reducing print resolution and fidelity [34,35].

Our rheological assessments of the hydrogels demonstrate increased storage (G′) and loss modulus (G″) values across all mixtures, indicating enhanced elasticity and robustness. Notably, the tan δ values, consistently below 1, confirm that these hydrogels exhibit a gel-like behavior rather than a liquid-like consistency, which supports their suitability for creating stable 3D structures. The viscosity profiles further reinforce the shear-thinning behavior, showing that viscosity is influenced by hydrogel composition (specifically dry content), temperature, shear force, and shear rate. These rheological traits are advantageous for extrusion bioprinting, as they minimize the likelihood of spreading while providing a protective matrix that could enhance cell viability. The individual components of the Alg-Gel hydrogel mixture contribute to distinct and complementary properties. Gelatin provides elasticity, improving cell adhesion within the hydrogel, while sodium alginate significantly contributes to the viscosity and stiffness of the scaffold [36,37]. Increasing Alg concentrations further enhances these mechanical properties, as reflected in the increase in both pore diameter and filament width. However, this must be balanced carefully, as higher viscosity and storage modulus due to increased Alg concentration can lead to higher extrusion pressures, potentially causing cellular damage [38].

Achieving high-resolution printed structures in extrusion bioprinting depends not only on material rheology but also on specific printer parameters, including nozzle diameter, printing temperature, printing pressure, and print speed. The nozzle diameter plays a critical role in controlling strand alignment and layer stacking during printing. Smaller nozzle diameters enable finer strand deposition, which is essential for achieving high-resolution structures, but this requires higher extrusion pressures to maintain consistent flow. These pressures can influence strand alignment, potentially causing misalignment or deformation, especially if the viscosity is too high. Additionally, small nozzle diameters may restrict the ability of the strands to stack coherently, leading to issues with layer adhesion and structural integrity [39]. On the other hand, larger nozzle diameters, while reducing the risk of nozzle clogging and cellular stress, may result in less precise alignment and reduced resolution, as the extruded strands are thicker and may spread more [40]. This compromise can impact the accuracy of the printed layers, leading to lower print fidelity. For instance, using smaller nozzle diameters with high extrusion pressures can enhance structural resolution but at the cost of increased cellular stress and damage. Conversely, larger nozzle diameters, while minimizing stress on cells, may compromise structural fidelity [41]. Through optimization of the Alg-Gel hydrogel mixture, our study identified that printing with a 203 μm inner diameter tapered nozzle allowed for a favorable balance, yielding high cell viability post-printing without compromising structure.

Previous studies have demonstrated that Alg-Gel hydrogels with Alg concentrations between 3 and 7% *w*/*v* and Gel concentrations of 6–8% *w*/*v* are conducive to biocompatibility and effective printability [42]. Our optimized blend, consisting of 7% alginate and 8% gelatin, was selected based on its ability to produce structures with satisfactory shape fidelity and cell viability immediately post-extrusion and after 15 days of culture. This blend exhibited promising potential for applications in bioprinting, where maintaining cell viability and structural fidelity is paramount.

Compared to previously reported bioinks, the Alg-Gel formulation described here demonstrates distinct advantages. For instance, widely used alginate-based bioinks often prioritize structural fidelity but struggle to support cell adhesion and proliferation due to their inert bioactivity. By incorporating gelatin into the formulation, this study addresses this limitation, as gelatin provides bioactive sites conducive to cell adhesion [43]. Additionally, other alginate–gelatin mixtures have reported lower storage modulus and shape fidelity when using lower alginate concentrations (<5%), whereas our formulation with 7% alginate strikes a balance between structural integrity and biocompatibility [44]. Moreover, while many bioinks require significant post-processing or complex crosslinking mechanisms to achieve stability, our formulation achieves robust mechanical properties and printing stability through simple ionic crosslinking, making it more accessible for diverse applications.

The rationale for selecting specific concentrations of alginate and gelatin in this study stems from the need to address key challenges in extrusion bioprinting. High alginate concentrations contribute to the structural integrity and mechanical strength of the scaffold, enabling the formation of consistent cylindrical fibers and coherent layer stacking. The nozzle diameter, printing pressure, and extrusion rate must be carefully optimized to ensure the proper alignment of these fibers and prevent uneven stacking, which could lead to weak spots in the printed structure [45]. However, excessive alginate can increase viscosity, requiring higher extrusion pressures that could adversely affect cell viability. The degradation of the hydrogel matrix over time may also contribute to a decline in cell viability, particularly after two weeks of culture. Hydrogel degradation, due to the ionic crosslinking mechanism, can lead to the release of degradation byproducts, which might interfere with cellular health [46]. Conversely, gelatin provides the necessary elasticity and bioactivity to support cell adhesion and promote viability within the hydrogel matrix. The combination of 7% alginate and 8% gelatin balances these competing demands by achieving adequate mechanical robustness, shear-thinning properties for smooth extrusion, and a matrix conducive to cellular growth and proliferation. This specific formulation also aligns with the observed rheological and mechanical characteristics, such as viscosity, storage modulus, and compressive modulus, which were found to be optimal for maintaining shape fidelity and supporting cell viability.

A noticeable decline in cell viability after two weeks was observed, which is likely attributable to hydrogel degradation rather than nutrient limitations, as no additional nutrient checks were conducted in this study. The hydrogel degradation over time may compromise the mechanical integrity of the scaffold and reduce the availability of essential bioactive sites for cell adhesion and proliferation [47]. Furthermore, the ionic crosslinking agents, such as calcium chloride, used to stabilize the alginate network could have potential cytotoxic effects on the cells. Residual calcium ions from the crosslinking process may contribute to cellular stress or interfere with normal cellular function. The significance of developing consistent 3D-bioprinted cancer models lies in their potential to replicate the complex tumor microenvironment. Traditional 2D cell culture models have long been used in preclinical drug discovery, yet they fall short in replicating in vivo conditions, which leads to limited translational success in drug efficacy testing. The emergence of 3D bioprinting offers a more sophisticated alternative, allowing for the formation of cellular spheroids within scaffold layers that mimic the native tumor microenvironment more accurately [48,49], such as the potential of the bioprinted tumor–vessel–bone co-culture model for preclinical drug testing, specifically by evaluating the sensitivity of the model to paclitaxel (PTX), a clinical drug used for BrCa treatment [50]. This model, by supporting spheroid formation in layered structures, opens new avenues for in vitro cancer research, particularly for drug screening applications, which could ultimately enhance the predictive value of preclinical tests.

Three essential properties guided the selection of Alg-Gel hydrogel mixtures for this study: (i) the ability to form consistent, cylindrical fibers; (ii) the capacity to stack printed layers coherently without spreading; and (iii) the need for low extrusion pressures to minimize cellular stress [51]. After testing various Alg-Gel ratios, four compositions were selected for 3D bioprinting, with each undergoing rigorous evaluation for shape retention and structural stability post-crosslinking. The 4% Alg–8% Gel composition demonstrated high viscosity, storage modulus, and loss modulus, resulting in a significantly higher compressive modulus. However, this formulation required a printing pressure of 100 psi, which is not conducive to cell viability due to excessive shear stress on cells during extrusion. In contrast, the 6% Alg–4% Gel formulation exhibited reduced viscosity, storage modulus, and compressive modulus, which adversely affected its shape fidelity post-printing. Maintaining structural integrity with this composition proved challenging. The formulations 5% Alg–6% Gel and 7% Alg–8% Gel showed comparable rheological and mechanical properties, including viscosity, storage modulus, and compressive modulus. However, the 7% Alg–8% Gel formulation demonstrated slightly superior characteristics and was easier to print using nozzles of varying gauges. This composition offered a balanced approach, providing adequate mechanical strength while maintaining favorable printability and shape fidelity.

Our findings highlight the intricate balance required between hydrogel rheological properties and printing parameters to achieve high-resolution, cell-compatible scaffolds in 3D bioprinting. The Alg-Gel hydrogel composition presented here offers a viable option for developing 3D-bioprinted cancer models that may advance preclinical drug testing by better simulating in vivo tumor characteristics.

## 5. Challenges and Future Directions

Despite the promising results demonstrated in this study, several challenges remain in advancing Alg-Gel hydrogel formulations for 3D bioprinting applications. The delicate balance between hydrogel viscosity, mechanical properties, and cell viability continues to pose a significant challenge. While increasing alginate concentration enhances mechanical strength, it often requires higher extrusion pressures that may compromise cell viability due to excessive shear stress. Conversely, reducing viscosity for better cell survival can undermine structural fidelity. Optimizing printing parameters, such as nozzle diameter, pressure, and speed, is critical to achieving high-resolution, cell-compatible scaffolds, but these parameters must be tailored for each hydrogel formulation. Additionally, the limited adhesion and proliferation observed with unmodified alginate formulations underscore the need for chemical modifications, such as incorporating cell-adhesion peptides, to improve cell–hydrogel interactions. Future directions should focus on integrating advanced materials and biofunctionalization strategies to enhance hydrogel biocompatibility and functional performance. The development of automated, real-time monitoring systems for extrusion parameters and cell viability could further improve reproducibility and efficiency. Moreover, leveraging computational modeling and machine learning to predict optimal hydrogel properties and printing conditions could significantly accelerate progress. Lastly, translating these advances into clinically relevant 3D-bioprinted cancer models will require robust validation to ensure their predictive value in drug screening and therapeutic testing.

## 6. Conclusions

This study highlights the potential of Alg-Gel hydrogels as versatile biomaterials for 3D bioprinting applications, particularly in the development of tumor models. Comprehensive rheological, mechanical, and biocompatibility assessments of various Alg-Gel formulations reveal the critical interplay between hydrogel composition and printing parameters. The optimized 7% Alg–8% Gel formulation demonstrated superior printability, structural fidelity, and cell compatibility, making it a promising candidate for extrusion-based bioprinting. The printability assessment of the 7% alginate–8% gelatin hydrogel revealed that the 27T tapered needle achieved the highest normalized Printability Index (POI_normalized_ = 1), demonstrating optimal performance with the narrowest strand width (0.56 ± 0.02 mm) and highest printing accuracy (97.2%) at the lowest printing pressure (30 psi). In contrast, the 30R needle, with the smallest inner diameter (0.152 mm) and highest printing pressure (80 psi), produced the widest strand width (0.70 ± 0.01 mm) and lowest accuracy (88.8%), resulting in a POI_normalized_ of 0.274. The 30T and 27R needles showed moderate performance, with POI_normalized_ values of 0.758 and 0.558, respectively. These findings highlight the significance of balancing needle geometry, printing pressure, and temperature for optimal hydrogel printability. Its shear-thinning behavior, tunable mechanical properties, and ability to support long-term cell culture underline its suitability for creating complex 3D scaffolds that mimic the native tumor microenvironment.

These findings underscore the importance of balancing hydrogel rheology, mechanical properties, and extrusion conditions to achieve high-resolution, cell-compatible bioprinted structures. The insights gained from this work provide a foundation for advancing 3D-bioprinted cancer models, offering a robust platform for preclinical drug discovery and personalized medicine. Future studies will focus on refining the hydrogel formulation and integrating multi-cellular co-cultures to further enhance the physiological relevance of these models.

## Figures and Tables

**Table 1:** Printing parameters for Alg-Gel hydrogel compositions in 3D bioprinting.

Hydrogel Composition	Needle Gauge (G)	Printing Temperature (°C)	Printing Pressure (psi)
4% Alg–8% Gel	30 R, 30 T, 27 R, and 27 T	25–35	30–80
5% Alg–6% Gel			
6% Alg–4% Gel			
7% Alg–8% Gel			

**Table 2:** Evaluation of 7% alginate–8% gelatin hydrogel printability across dispensing needle sizes and printing pressure ranges.

Needle Gauge (G)	Inner Diameter (mm)	Printing Pressure (psi)	Strand Width (mm)	Printing Accuracy (%)	POI_normalized_
30 R	0.152	80	0.70 ± 0.01	88.8	0.274
30 T	0.150	35	0.63 ± 0.01	96.0	0.758
27 R	0.200	50	0.57 ± 0.02	92.0	0.558
27 T	0.203	30	0.56 ± 0.02	97.2	1.000

## Data Availability

No new data were created or analyzed in this study. Data sharing is not applicable to this article. All data could be found in the main text.
